# Supplementary material for: Camellia sinensis Chloroplast Fluoride Efflux Gene CsABCB9 Is Involved in the Fluoride Tolerance Mechanism
Source: Int J Mol Sci. 2022 Jul 14;23(14):7756. doi: 10.3390/ijms23147756 (PMC9317437; doi:10.3390/ijms23147756)
Supplement: Supplementary file 1 [file ijms-23-07756-s001.zip › ijms-1794412-supplementary.pdf]

Table S1. ABC transporter gene information analyzed by transcriptome.

Items	Number
ABC transporter A family	53
ABC transporter B family	131
ABC transporter C family	118
ABC transporter D family	16
ABC transporter E family	3
ABC transporter F family	36
ABC transporter G family	79
ABC transporter I family	12

Table S2. Differentially expressed genes of the transporter family were analyzed from the transcriptome database.

Family	Up number	Maximum expression	Genes ID	Down number	Minimum expression	Genes ID
ABCB	3	3.89	CL667.Contig7_All	11	-3.4	Unigene13955_All
ABCC	3	2.6	CL12464.Contig8_All	3	-1.6	CL9013.Contig3_All
ABCD	0	2.59	CL1487.Contig2_All	0	-1.20	CL1487.Contig3_All
ABCE	0	1.26	CL11662.Contig2_All	0	-0.92	Unigene53555_All
ABCF	1	2.3	Unigene44093_All	2	-1.7	CL22135.Contig1_All
ABCG	0	1.96	Unigene76236_All	5	-3.6	CL2493.Contig4_All
ABCI	0	0.25	Unigene17826_All	0	-0.21	Unigene134470_All

Table S3. Primers designed in this study.

Primer name	Sequence (5'-3')	Annotation
CsABCB9-PET28a-F	ATGGGTCGCGGATCCGAATTCATGGCTCTTCCTCTCTGCGAT	construction of the PET28a
CsABCB9-PET28a-R	TTGTCGACGGAGCTCGAATTCTCATTCACTGTGATGCATTATTGAGG	
CsABCB9-GFP-F	AAGTCCGGAGCTAGCTCTAGAATGGCTCTTCCTCTCTGCGAT	construction of the subcellular localization vectors
CsABCB9-GFP-R	GGTCCTCGAGACGTCTCTAGATTCACTGTGATGCATTATTGAGGA	
GFP-CsABCB9-F	ACGGCATGGACGAGCTGTACAGATCT ATGGCTCTTCCTCTCTGCGAT	
GFP-CsABCB9-R	GCAGCCGGGCGGCCGCTTTAAGATCTTCATTCACTGTGATGCATTAT	
CsABCB9-pT7Ts-F	GGCAGATCTGATATCACTAGTATGGCTCTTCCTCTCTGCGAT	construction of the pT7Ts
CsABCB9-pT7Ts-R	CGATCCTAGTCAGTCACTAGTTCATTCACTGTGATGCATTATTGAGG	
CsABCB9-qPCR-F	ACCACTACCACCACCACAAA	qRT-PCR
CsABCB9-qPCR-R	GATGGTCTGCGTCGGAAATC	
CsGAPDH-qPCR-F	GGCAGCACCTTACCAACAGC	internal reference gene in *C.sinensis*
CsGAPDH-qPCR-R	GTTTGGCGTCGTTGAGGGTC	
CsFEX-qPCR-F	GGTAGTTGTATTCACCGC	qRT-PCR
CsFEX-qPCR-R	GCATTGTTGGACCTTTCG	
AtACTIN2-F	CTCCCGCTATGTATGTCGCC	internal reference gene in *A. thaliana*
AtACTIN2-R	TTGGCACAGTGTGAGACACAC	
CsABCB9-RT-PCR-F	GCTCTTCCTCTCTGCGATCT	identification of positive *A. thaliana* lines
CsABCB9-RT-PCR-R	TTTGTGGTGGTGGTAGTGGT	

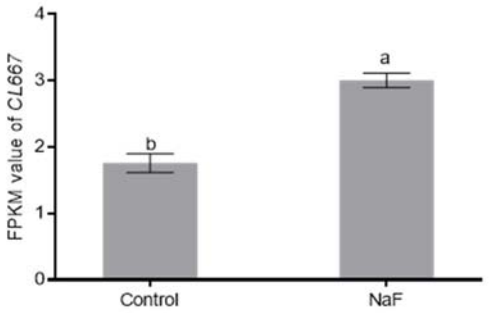

Figure S1. The FPKM (Fragments per kilo base per million mapped reads) values of *CL667* under F^- treatment obtained from RNA-Seq: Data are shown as mean ± SD (n=2 plants). Different letters indicate significant differences between treatments (P<0.05, one-way ANOVA).

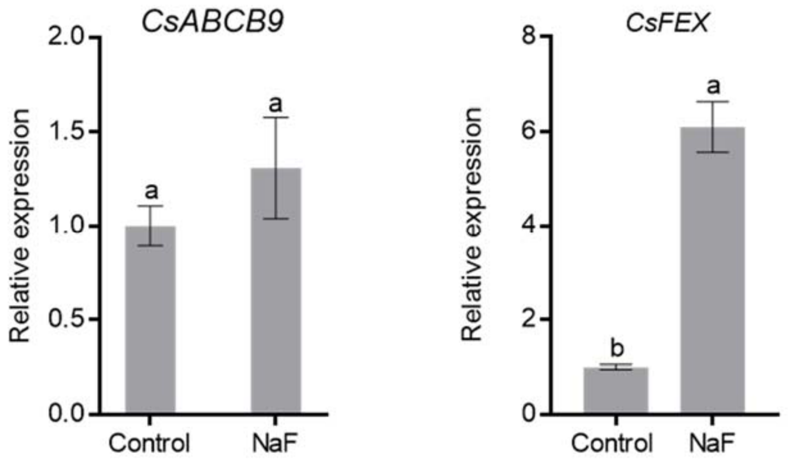

Figure S2. Expression patterns of *CsABCB9* and *CsFEX* in tea plant roots under

F^- treatments: Data are shown as means ± SD (n = 4). Gene expression values under control conditions were set as 1. Different letters indicate significant differences between treatments ($P < 0.05$, one-way ANOVA).

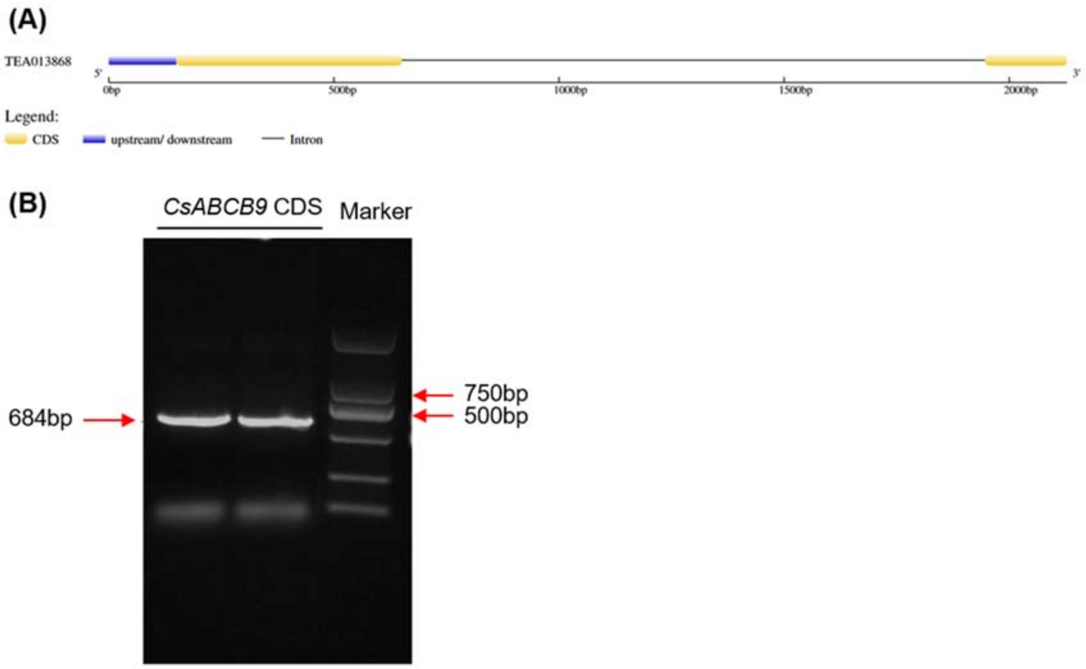

Figure S3. Genetic structure and cloning of the *CsABCB9*: (A) Structural characteristics of the *CsABCB9* gene by Gene Structure Display server (http://gsds.gao-lab.org/), (B) Cloning of *CsABCB9* CDS sequence from tea leaves cDNA.

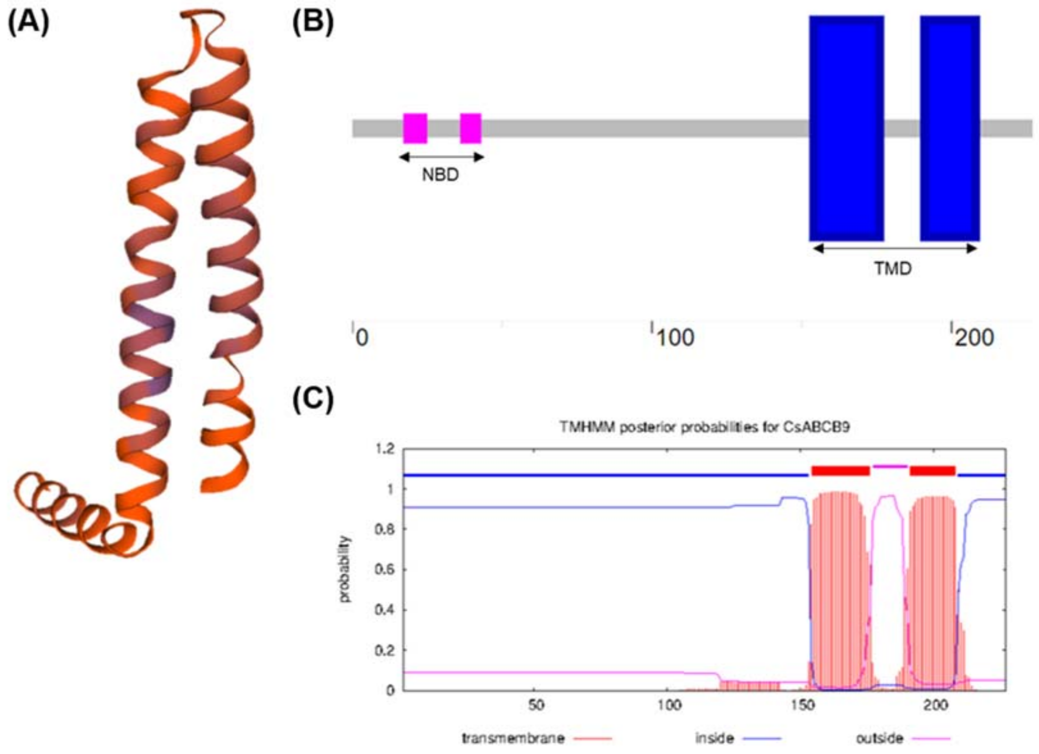

Figure S4. Structural characteristics of CsABCB9 protein: (A) predicted tertiary structure of CsABCB9 protein by SWISS-MODEL (https://swissmodel.expasy.org/), (B) SMART (Simple Modular Architecture Research Tool) analysis (http://smart.embl-heidelberg.de/) of the conserved domains of CsABCB9 protein, (C) transmembrane topology model of CsABCB9 protein is predicted by TMHMM (https://services.healthtech.dtu.dk/).
